# Longitudinal stability of cognitive impairments in post-COVID-19 syndrome assessed with the tablet-based Oxford Cognitive Screen-Plus

**DOI:** 10.1038/s41598-026-48476-5

**Published:** 2026-04-16

**Authors:** Valeska Kozik, Philipp A. Reuken, Katzer Katrin, Zoe Stallmach, Isabelle Utech, Nele Demeyere, Matthias Schwab, Andreas Stallmach, Kathrin Finke

**Affiliations:** 1 Department of Neurology, Jena University Hospital/Friedrich-Schiller-University, Jena, Germany; 2 Department of Internal Medicine IV (Gastroenterology, Hepatology and Infectious Diseases), Jena University Hospital/Friedrich-Schiller-University, Jena, Germany; 3 Nuffield Department of Clinical Neurosciences, University of Oxford, Oxford, United Kingdom; 4 Department of Psychology, Ludwig-Maximilians-Universität München, Munich, Germany

**Keywords:** Post-COVID-19 Syndrome, Cognitive Dysfunction, Neuropsychological Assessment, Longitudinal Studies, Digital Health, Diseases, Health care, Medical research, Neurology, Neuroscience, Psychology, Psychology

## Abstract

In a previous cross-sectional study using the tablet-based Oxford Cognitive Screen-Plus (OCS-Plus), deficits in delayed memory, attention, and executive functioning were identified in working-age patients with post-COVID-19 syndrome (PCS) following infection in 2020 or early 2021. Initial assessment occurred approximately five months after infection. To examine short-term longitudinal trajectories, patients were reassessed several months later. Eighty-one patients with PCS (mean age 46.6 years, 64% female) completed OCS-Plus assessments at baseline and after a median follow-up of 4.4 months. Cognitive change was analysed using Wilcoxon signed-rank tests and equivalence testing (± 1 SD of reference scores) to assess clinical relevance. Associations between cognitive change and changes in depression and fatigue were examined using bootstrap-corrected multiple regression. No significant change in cognitive performance was observed between baseline and follow-up across any cognitive domain (all *p* > 0.3). Equivalence testing indicated that observed differences fell within predefined bounds of clinical insignificance (all *p* < 0.01). Changes in depressive symptoms and fatigue were not associated with changes in cognitive performance. Across the observed follow-up period, domain-level cognitive performance remained stable, with no evidence of short-term spontaneous improvement. These findings suggest short-term longitudinal stability of cognitive impairments in PCS within the limits of screening-based assessment and the follow-up interval studied, supporting the value of continued cognitive monitoring in affected individuals.

**Supplementary Information:**

The online version contains supplementary material available at 10.1038/s41598-026-48476-5.

## Introduction

Post-COVID-19 syndrome (PCS) refers to persistent symptoms lasting for at least three months after acute SARS-CoV-2 infection that cannot be explained by an alternative diagnosis^1^. Among the most disabling symptoms are cognitive deficits, often referred to as “brain fog”, which primarily manifest as deficits in memory, attention, and executive functioning^2,3^. These cognitive impairments are associated with reduced quality of life and may interfere with occupational reintegration and everyday functioning^4^. Objective cognitive deficits in PCS have now been demonstrated across multiple cohorts and study designs^5^. However, longitudinal evidence remains limited and methodologically heterogeneous, with substantial variation in patient selection, timing of assessment, and cognitive measurement approaches. The natural course, i.e., whether cognitive impairments resolve spontaneously, persist, or worsen, remains underexplored. While some longitudinal studies report partial improvement in specific domains over time, others describe persistent deficits over months to years, and direct comparisons are hampered by differences in baseline assessment timing and sample composition (e.g^6–9^). A further limitation of the existing literature is potential selection bias: many studies assess patients who already have prolonged or severe PCS at their first cognitive evaluation, which may systematically exclude individuals whose cognitive symptoms resolved earlier and thereby overrepresent persistent impairment. This limits conclusions about the broader natural course of cognitive recovery in PCS.

An additional challenge in PCS research concerns the measurement of cognitive dysfunction itself. Recent clinic-based studies have highlighted that commonly used global cognitive screening instruments may insufficiently capture the attentional, executive, and processing-speed deficits frequently reported in PCS, whereas brief, domain-targeted assessments appear more sensitive to this cognitive profile^10^. This has contributed to ongoing discussion regarding the appropriate balance between assessment depth and feasibility when evaluating cognitive impairment in PCS within routine clinical settings.

In our previous study^11^ we examined patients who had contracted SARS-CoV-2 early in the pandemic, before vaccines were available. These patients were infected early in the pandemic, prior to vaccine availability, during a period when wild-type, alpha, and delta variants predominated. They were assessed using the tablet-based Oxford Cognitive Screen-Plus (OCS-Plus^12^), a brief digital cognitive assessment designed to capture domain-specific cognitive performance with standardised administration and minimal training requirements. That study identified impairments in delayed memory, attention, and executive functioning compared with healthy reference data. These findings were consistent with results from studies employing more extensive neuropsychological batteries, supporting the utility of brief, domain-focused digital tools for detecting clinically relevant cognitive domain-level deficits in PCS^13,14^. However, the cross-sectional design precluded conclusions regarding the temporal stability or recovery of these impairments.

The present study addresses this gap by providing a longitudinal follow-up of a well-characterised PCS cohort whose baseline cognitive assessment occurred approximately five months after infection, i.e., early in the course of the syndrome. By reassessing cognitive performance after several months using the same standardised digital tool, we aimed to examine short-term longitudinal trajectories of domain-level cognitive performance and to determine whether spontaneous recovery could be observed within this clinically relevant timeframe. This study aims to determine whether spontaneous recovery occurs or whether persistent deficits necessitate targeted interventions. Cognitive performance was assessed using the OCS-Plus, a brief, validated digital tool sensitive to subtle, domain-specific impairments^12^. In addition, given the high prevalence of fatigue and depressive symptoms in PCS and their potential influence on cognitive performance, we examined whether changes in fatigue or depressive symptoms were associated with changes in cognitive performance over time.

## Methods

### Research design, participants, and recruitment

In this observational, longitudinal study, patients with PCS were assessed in the post-COVID-19 outpatient clinic at Jena University Hospital (Germany). For the initial assessment, we included 282 patients who visited the clinic between August 2020 and March 2022^11^. All patients had confirmed positive results for SARS-CoV-2 through PCR testing, provided informed consent, and met the diagnostic criteria for PCS, meaning that their symptoms had persisted for at least three months after acute infection without an alternative explanation. Additional inclusion criteria were age 18–65 years, no known relevant neurological or severe psychiatric disorders that could impact cognition, no significant vision or hearing problems interfering with cognitive testing, and sufficient German language skills for test administration.

Follow-up cognitive assessments were initially planned for all participants during their second clinical visit using the same standardised protocol administered at baseline. Follow-up testing was discontinued due to institutional constraints (e.g., limited resources, staffing reassignments), rather than patient-related factors. This externally imposed cut-off reduces the likelihood that follow-up participation was contingent on symptom persistence or perceived recovery. As a result, 81 participants (29% of the baseline cohort) completed follow-up assessments at a median of 4.4 months post-baseline. To evaluate the potential for attrition-related bias, baseline sociodemographic, clinical, and cognitive variables were compared between participants who completed follow-up and those who did not. In addition, effect sizes with 95% confidence intervals were calculated. These comparisons included age, sex, baseline cognitive performance across all domains, hospitalisation and ICU treatment during acute COVID-19, and baseline fatigue and depressive symptoms. No statistically significant group differences were observed on any of these measures (all *p* > 0.05). Effect sizes were small, and confidence intervals either spanned or were close to zero, reducing concern that follow-up participation was systematically driven by baseline clinical severity or cognitive impairment. During the clinical routine assessment, a structured history was taken from each patient at baseline, including medical history, sociodemographic data, and subjective complaints. Mood and fatigue were assessed via standardised questionnaires at both visits:

- The Patient Health Questionnaire (PHQ-9) depression module for depressive symptoms^15^. Scores ≥ 5 indicate clinically relevant depressive symptoms.
- Fatigue Assessment Scale (FAS) for subjective fatigue^16^. Scores > 21 indicate fatigue and those > 34 indicate severe fatigue^17^.

See Table 1 for full cohort characterisation.

**Table 1:** **Study cohort medical history, sociodemographic characteristics and follow-up**

	**Distribution**	
**Baseline sociodemographic data (visit 1)**		
Sex, no. (%), female / male	52 / 29	(64 / 36)
Age, years, mean (SD, range)	47.6	(10.4, 22–64)
Education, years, mean (SD, range)	14	(2, 10–18)
**Medical history and COVID-19 infection**		
*All patients (n = 81)*		
Comorbidities		
Cardiovascular diseases, No. (%)	35	(43.2)
Diabetes mellitus, No. (%)	5	(6.2)
Psychiatric comorbidities, No. (%)	10	(12.3)
Months between infection and visit 1, median (Q1, Q3, range)	5.4	(4.3, 6.6, 2.8–14.9)
Hospital admission, No. (%), yes / no	24 / 57	(29.6 / 70.4)
Year of infection, No. (%), 2020 / 2021	58 / 23	(71.6 / 28.4)
Vaccinated at visit 1, No. (%), yes / no	0 / 81	(0 / 100)
*Hospitalised patients (n = 24)*		
ICU ward, No. (%)	8	(33.3)
Hospital stay, median (Q1, Q3), days	9	(7, 14.3)
ICU stay, median (Q1, Q3), days	6	(2.5, 10)
Oxygen support, No. (%)	17	(70.8)
**Follow-up timeline**		
Months between visits, median (Q1, Q3, range)	4.4	(3.9, 4.6, 3.2 –9.6)
**Mood and fatigue**		
*Visit 1*		
FAS, fatigue, raw score, mean (SD, range)	31	(8.6, 13–50)
PHQ-9, depression, raw score, mean (SD, range)	10.7	(4.5, 1–23)
*Visit 2*		
FAS, fatigue, raw score, mean (SD, range)	29.8	(8.6, 12–48)
PHQ-9, depression, raw score, mean (SD, range)	9.4	(4.5, 1–20)

Note. SD = standard deviation; Q1, Q3 = quartiles 1, 3; ICU = intensive care unit; FAS = Fatigue Assessment Scale; PHQ-9 = Patient Health Questionnaire, depression module.

### Cognitive assessment

Cognitive functioning was assessed using the computerised OCS-Plus, a validated tool for detecting subtle cognitive impairment^12^. Administered via stylus on a tablet, the OCS-Plus takes approximately 25 min to complete and begins with tasks evaluating orientation, picture naming, and semantic understanding to assess basic cognitive status. In patients with PCS, normal performance on these tasks helps rule out generalised impairment, indicating that any additional deficits are domain-specific. Subsequent tasks assess core cognitive domains, including memory, attention, executive functioning, and praxis. Specifically, memory tasks examine encoding efficiency and delayed retrieval; executive tasks measure shifting accuracy and inhibitory control; praxis tasks assess visuomotor integration and figure reproduction; and attention tasks target selective attention using visual cancellation.

From nine OCS-Plus subtasks, six composite scores were computed:

- Naming and Semantic Understanding (Picture Naming + Semantics).
- Memory Encoding (Encoding 1 + Encoding 2).
- Delayed Memory (Delayed Recall + Delayed Recall and Recognition).
- Praxis (Figure Copy + Figure Recall).
- Attention (Cancellation + Invisible Cancellation).
- Executive Functioning (Trails Executive Score – Cancellation false positives).

For complete task descriptions, see supplementary materials, Table S1. Testing was conducted under standardised conditions, with patients seated in a quiet room using a 10-inch touchscreen tablet.

Due to technical issues during the second tablet-based assessment (e.g., input registration), which were unrelated to participants’ cognitive performance or clinical status, some composite scores (Executive Functioning and Praxis) had slightly smaller sample sizes (see Table 2).

### Statistical analysis

Each analysis was conducted using all available cases, with no imputation performed. Statistical significance was set at a threshold of *p* < 0.05.

## Basic cognitive status tasks

Descriptive statistics were computed for basic orientation and the Naming and Semantic Understanding composite to assess fundamental cognitive capacity. Due to limited variability, i.e., ceiling effects, no statistical analyses were conducted on these scores (see results section).

## Group-level change

**Fig. 1:**
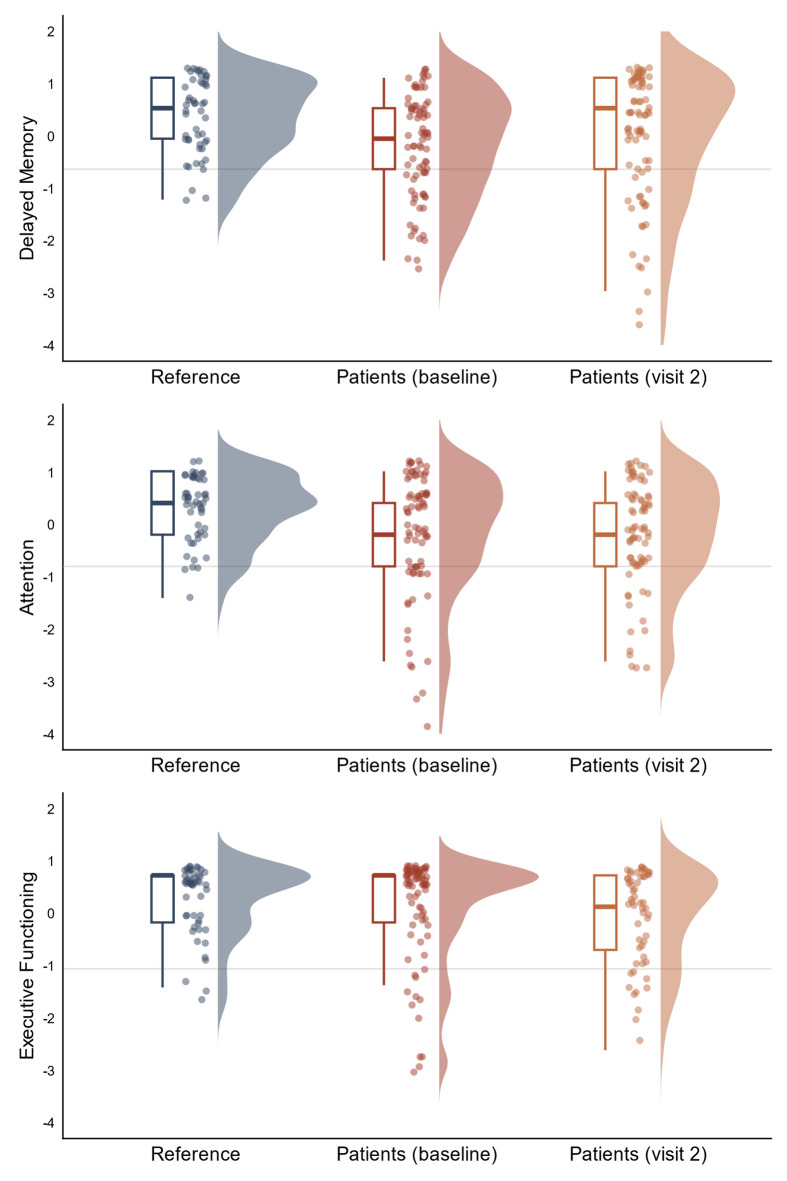
Follow-up patients’ performance on initially impaired OCS-Plus cognitive domains. *Note*. Displayed are z-transformed performance scores for the three cognitive domains impaired at baseline. Depicted are standard boxplots, individual performance (dots), and density curves. The horizontal line represents the cut-off based on healthy reference performance at baseline^11^. The figure includes all healthy references and patients with data from two visits.

To assess changes in cognitive performance between baseline and follow-up, we analysed differences in the OCS-Plus composite scores, covering five cognitive domains (Memory Encoding, Delayed Memory, Praxis, Attention, and Executive Functioning). Given the non-normal distribution in some subtests and the potential sensitivity to extreme values, we used paired Wilcoxon signed-rank tests (*stats*, v4.4.1), a non-parametric method that takes into account skewed distributions. Applying conservative corrections for multiple comparisons (FDR) did not alter any conclusions, as all p-values remained above 0.05. In Fig. 1, to facilitate direct comparison between visits as well as with healthy references, patient performance at visit 2 was z-transformed according to data from the cross-sectional study^11^. Proportional change in performance was calculated as (*performance at visit 2 – performance at baseline*)/*baseline performance* (see Table 2).

### Magnitude and consistency of change

To quantify the magnitude and consistent direction of change between visits, we computed the rank-biserial correlation (*rcompanion*, v2.4.36) for each core cognitive domain. The correlation was based on the difference between visit 2 and baseline scores, where positive values indicate improvement. The rank-biserial correlation is used as a non-parametric measure of effect size, quantifying both the strength and direction (improvement or decline) of individual patients’ change in cognitive performance. A positive rank-biserial correlation coefficient (*rc* > 0) indicates consistent improvement, a negative coefficient (*rc* < 0) indicates consistent decline, and *rc* ≈ 0 indicates no directional change. Confidence intervals for the rank-biserial correlations were computed using bias-corrected and accelerated (BCa) bootstrap resampling with 1000 iterations, with significance determined by 95% CIs not overlapping zero. Sensitivity analysis indicated that with *N* = 81, we had approximately 80% power to detect small-to-moderate directional effects (corresponding to rank-biserial correlation rb ≈ 0.15) using a two-sided paired comparison at α = 0.05. Smaller effects (e.g., rb ≈ 0.10) may have gone undetected.

To formally assess whether paired differences in each cognitive domain are clinically negligible, we implemented an equivalence testing approach using the two one-sided tests (TOST) procedure (*TOSTER* v0.8.4)^18^. Equivalence bounds were defined on the raw scale as ± 1 SD of reference scores, reflecting a conservative benchmark for clinically meaningful change commonly used in neuropsychological research and clinical practice, when validated functional anchors are unavailable. Any change smaller than 1 SD can be considered not clinically relevant^19^. In view of deviations from normality in the difference scores, we employed a bootstrapped TOST with 1000 replications and studentized 95% CIs. Equivalence was concluded if both one‐sided tests yielded p-values < 0.05 and if the 90% confidence interval for the mean difference, corresponding to conducting two one-sided tests at α = 0.05, was entirely contained within the prespecified bounds.

To address variability in follow-up intervals, associations between time between assessments and cognitive change scores were examined using Spearman correlations with bootstrapped confidence intervals (BCa, 1000 iterations).

### Exploratory analyses

To examine whether changes in cognitive performance were associated with changes in fatigue and depression, linear regression models were fitted for each cognitive domain initially impaired. Given non-normal residuals, CIs were computed using BCa bootstrap resampling with 1000 iterations.

### Software

Data preparation, analysis, and visualisation was performed using R version 4.4.3.

### Ethics

The study was conducted in accordance with the Declaration of Helsinki and was approved by the ethics committee of the Jena University Hospital (amendment to 5082-02/17). Written, informed consent was obtained from all participants before study enrolment.

## Results

### Basic cognitive status tasks

Performance on the orientation and Naming and Semantic Understanding tasks showed significant ceiling effects. At baseline, all patients (100%) made one or fewer errors on the orientation task, with 98.8% maintaining this at the second visit. Similarly, 98.7% of patients made one or fewer errors on the Naming and Semantic Understanding composite at baseline, and 100% did so at follow-up. No patients made errors on both measures at baseline, and only one patient did so at follow-up, though their performance remained within the clinically unimpaired range. No deficits indicative of insufficient comprehension or potential neurodegenerative impairment were observed.

### Group-level change

There was no evidence of change in performance on any of the OCS-Plus cognitive domain composite scores (all *p* > 0.05, see Table 2). Figure 1 depicts z-transformed performance on the OCS-Plus composite scores. The figure depicts performance in healthy references (data from previous cross-sectional study)^11^ as well as baseline and follow-up performance in FU patients.

### Magnitude and consistency of change

For cognitive domains where performance was initially impaired compared to healthy reference participants, the relative change was small. The median change scores at baseline for these domains were close to zero, with interquartile ranges indicating minimal variation (Table 2). Similarly, for domains with normal baseline performance, the relative change in performance remained minimal, with median change scores also close to zero (Table 2).

**Fig. 2:**
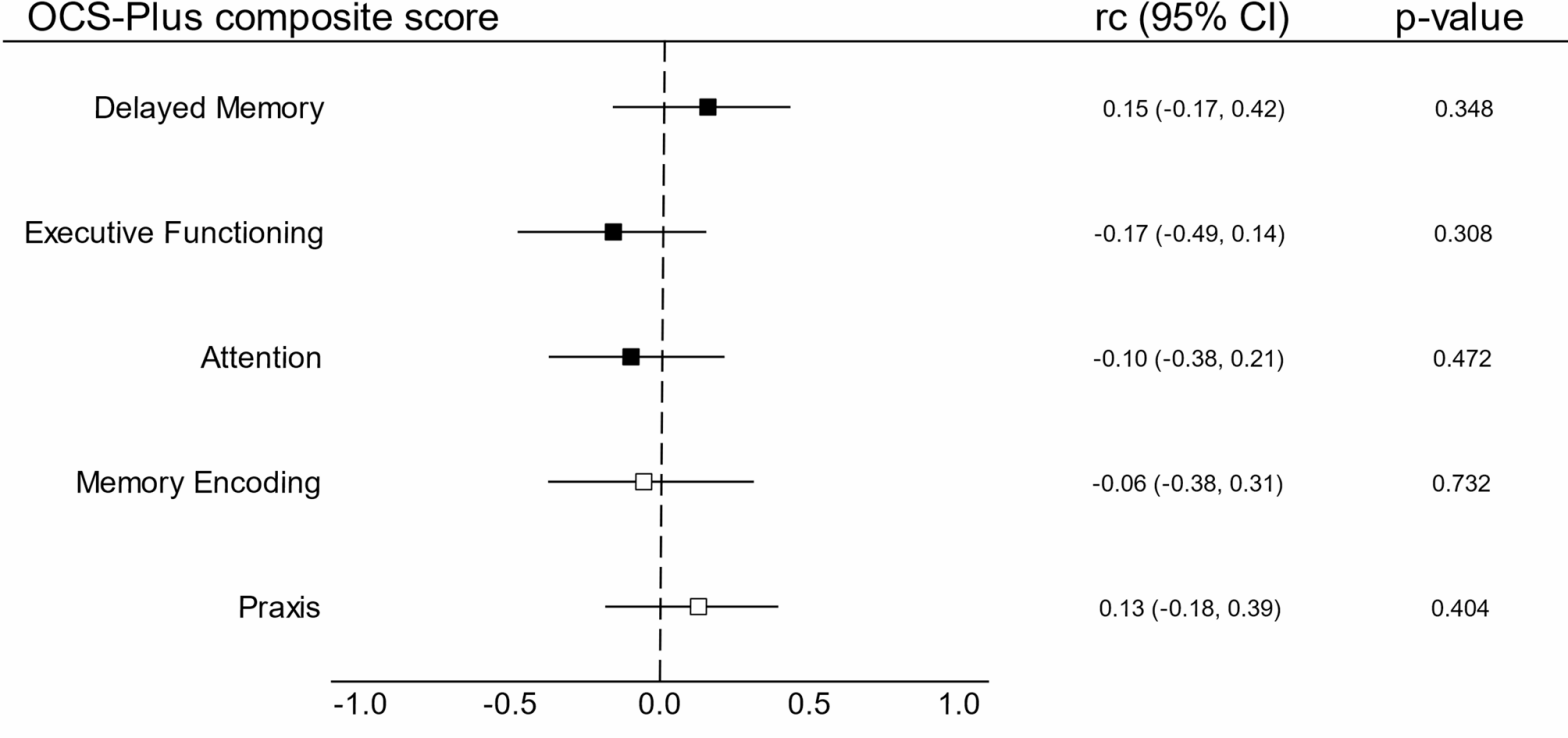
Relative change from baseline per cognitive domain on the OCS-Plus in PCS patients. Note. Forest plot of effect sizes (*rc* = rank-biserial correlation coefficient) with 95% confidence intervals (CI) for Oxford Cognitive Screen-Plus (OCS-Plus) composite scores. Effect sizes in the middle, and 95% CI with *p*-values on the right. The dashed line at zero denotes no effect (*rc* = 0). Filled boxes indicate composite scores impaired at baseline, empty boxes indicate scores unimpaired at baseline.

Figure 2 shows rank-biserial correlations of OCS-Plus composite score performance of FU patients between visits. The rank-biserial correlations for the five core cognitive domains were generally small and none reached statistical significance, as all CIs included zero.

Using the TOST procedure, with equivalence bounds set at ± 1 SD of reference scores, all domains tested showed equivalence, with all *p* < 0.01 (see Table 2), which suggests stability of performance across visits.

Spearman correlations between follow-up interval and domain-specific cognitive change were small and non-significant across all domains (rho = − 0.21 to 0.15), with bootstrapped confidence intervals spanning zero, providing no evidence for a systematic association between reassessment timing and cognitive change.

Overall, we found no evidence for a directional change in performance between visits in this cohort.

### Exploratory analyses

Changes in fatigue and depression were not found to be associated with changes in cognitive performance, with confidence intervals consistently including zero (for full results, see supplementary materials, Table S4).

**Table 2:** Change in OCS-Plus composite score performance from baseline to assessment two.

		Proportional change		Comparisons			
				WSR		TOST	
Composite score	*n*	Q2	(Q1, Q3)	V	p	Lower p	Upper p
*Performance initially impaired compared to reference*							
Delayed Memory	81	0	(–0.1, 0.2)	820	0.348	< 0.001	< 0.001
Executive Functioning	55	–0.1	(–0.2, 0.2)	510	0.308	< 0.001	0.001
Attention	77	0	(0, 0)	846.5	0.472	0.001	< 0.001
*Performance initially normal compared to reference*							
Memory Encoding	78	0	(–0.1, 0)	367	0.732	< 0.001	< 0.001
Praxis	58	0	(0, 0.1)	931.5	0.404	< 0.001	< 0.001

Note. WSR = Wilcoxon signed-rank test; TOST = two one-sided tests procedure for equivalence testing; Lower p = p-value for testing if the observed difference is greater than the lower equivalence bound (−1 SD); Upper p = p-value for testing if the observed difference is smaller than the upper equivalence bound (+ 1 SD); Q1, 2, 3 = quantiles 1, 2, 3; Proportional change is defined as *performance at visit 2 – performance at baseline*)/*baseline performance*. For a composite score, equivalence between visits is considered significant if both p-values (lower and upper bounds) are < 0.05, indicating that the observed change falls within the predefined equivalence bounds; Composite scores are grouped by impairment at baseline, compared to healthy reference participants.

## Discussion

In this longitudinal study, patients with PCS who were initially assessed with the tablet-based OCS-Plus at a median of approximately five months post-infection, and in whom impairments were observed in attention, delayed memory and executive functioning performance compared to healthy references^11^, were reassessed at a median of approximately four months later. The findings indicate that domain-level cognitive performance in patients with PCS remained stable in the short- to medium-term, with no evidence of spontaneous improvement within the observed timeframe. Across the assessed cognitive domains, performance remained within the same clinical range between assessments. For the previously impaired domains of delayed memory, executive functioning, and attention, performance remained within the same clinical range. For domains without baseline impairments, performance continued to fall within normative limits. Exploratory analyses provided no evidence that variability in follow-up interval was systematically related to cognitive change, although subtle timing effects cannot be excluded given the sample size and variability in reassessment intervals. Taken together, these findings indicate short-term longitudinal stability of cognitive impairments in PCS at the level captured by standardised cognitive screening, with no evidence of spontaneous improvement within the observed follow-up period. However, the present follow-up duration does not allow conclusions regarding longer-term recovery or deterioration beyond the interval studied. Changes in depression or fatigue were not observed to relate to the cognitive difference score between visits, suggesting that cognitive impairments in PCS may show short-term stability that is independent of concurrent changes in mood or fatigue.

A key strength of our study is its unique patient cohort, consisting of patients who contracted SARS-CoV-2 in 2020 or spring 2021, before vaccines were available to them. As all patients were unvaccinated at the time of infection, this provides a rare opportunity to examine the natural course of PCS-related cognitive impairments without the potential modifying effects of prior immunisation. Importantly, the initial cognitive assessment with the OCS-Plus was conducted shortly after patients met the diagnostic criteria for PCS. By capturing this early syndrome phase, our study provides a more representative perspective on the early natural course of cognitive impairments in PCS and minimises the risk of systematically excluding patients who may have recovered earlier.

At the same time, the clinical setting of recruitment should be considered when interpreting these findings. Participants were recruited from a specialised tertiary post-COVID clinic and therefore represent a subgroup of patients with a higher burden of acute illness and persistent symptoms than the broader community-based PCS population. As such, the present findings may not be directly generalisable to patients with post-COVID symptoms which do not require specialised care. However, as this cohort may represent individuals at higher risk for prolonged impairment, the observed absence of spontaneous cognitive improvement over the follow-up period is particularly relevant and highlights the need for targeted monitoring in this subgroup.

As these earlier cases are no longer able to be recruited, our study provides a unique opportunity to examine the long-term cognitive consequences in patients affected by the initial, more aggressive phase of the pandemic. Future studies should consider how changes in viral variants, vaccination status, and early treatment interventions may alter the trajectory of cognitive impairment in PCS. A comparison of cohorts from different pandemic phases will be essential to distinguish the specific impact of SARS-CoV-2 infection itself from potential modifying factors such as immune response differences, hybrid immunity, and medical interventions.

The observed short-term stability of deficits in attention, memory, and executive functioning over the follow-up period raises questions about their functional relevance during the early to medium phase of PCS. Although real-world outcomes such as occupational performance or daily functioning were not directly assessed, cognitive functions in these domains are known to be relevant for cognitively demanding activities. Importantly, the absence of measurable improvement within the observed timeframe provides no evidence for spontaneous cognitive recovery in the early to medium phase of PCS. These findings underscore the potential value of cognitive monitoring in clinical follow-up, particularly to identify individuals who may benefit from further assessment or supportive interventions. However, conclusions regarding longer-term functional consequences, employability, or recovery trajectories beyond the studied interval remain premature and require confirmation in studies with extended follow-up and direct functional outcome measures.

A key limitation of this study is the reduced follow-up sample, which limits statistical power and generalisability. To address potential attrition bias, we compared participants who completed follow-up with those who did not across baseline sociodemographic, clinical (hospitalisation, ICU treatment, fatigue, depression), and cognitive variables. No significant group differences were observed on any of these measures, reducing concern that follow-up participation was systematically driven by baseline clinical severity or cognitive impairment. Nevertheless, the possibility of unmeasured factors influencing follow-up participation cannot be fully excluded. Although follow-up assessments were discontinued due to externally imposed clinical workload constraints rather than patient dropout from clinical care, it cannot be excluded that patients who experienced subjective improvement were less likely to participate in the research reassessment. Secondly, the follow-up interval was relatively short, with a median of approximately four months between assessments. While this timeframe may not capture longer-term or delayed changes, it reflects a clinically relevant window in which recovery might typically be expected. The absence of measurable improvement during this period provides no evidence for spontaneous resolution of deficits and thus warrant structured follow-up and cognitive monitoring in patients with PCS beyond the initial diagnostic visit. Additionally, the equivalence bounds were based on a conservative distribution-based threshold rather than PCS-specific validated anchors; alternative thresholds may yield different conclusions, and this should be considered when interpreting the equivalence findings. Thirdly, while baseline analyses indicated an association between acute disease severity and cognitive performance, no evidence of change in cognitive performance was observed between baseline and follow-up in the present study. The extent to which disease severity influences longitudinal cognitive trajectories therefore remains uncertain, and generalisability to community-based PCS populations with milder symptom profiles may be limited. Lastly, the OCS-Plus is a screening instrument rather than a comprehensive neuropsychological battery. While it is designed to capture clinically relevant impairments at the domain level, more fine-grained cognitive heterogeneity within domains cannot be assessed using this approach. Although the OCS-Plus is well validated in other neurological populations and was selected for its sensitivity to domain-specific impairments relevant to PCS, it has not yet been formally validated specifically in post-COVID-19 syndrome, which represents an additional limitation. Moreover, alternate forms and task randomisation were not available, and practice effects therefore cannot be excluded; such effects may bias results towards apparent stability and could obscure subtle cognitive deterioration over time. Finally, as a tablet-based assessment, performance may be influenced by factors such as digital familiarity or fine motor coordination, which could affect test results independently of cognitive function, particularly across a broad age range.

## Conclusions

In this longitudinal study, no evidence of short-term spontaneous improvement was found, as no changes were observed in cognitive performance in patients with PCS across the follow-up period. For domains that were initially impaired, such as delayed memory, executive functioning, and attention, performance remained within the same clinical range, suggesting that cognitive impairments in PCS may remain stable over the short term, independent of mood or fatigue fluctuations. These findings highlight the importance of neuropsychological monitoring and timely intervention, especially in patients potentially at risk of longer-term cognitive impairment. As this cohort was infected during the early phase of the pandemic, prior to vaccination and therapeutic interventions, future studies should examine how factors such as vaccination status, viral variant, and early treatment influence cognitive outcomes in more recent PCS populations.

## Supplementary Information

Below is the link to the electronic supplementary material.

Supplementary Material 1

## Data Availability

De-identified data supporting this study may be shared based on reasonable written requests to the corresponding author.

## Declarations

### Brief title

Stability of Cognitive Deficits in Post-COVID-19.
