# Supplementary material for: Longitudinal stability of cognitive impairments in post-COVID-19 syndrome assessed with the tablet-based Oxford Cognitive Screen-Plus
Source: Sci Rep. 2026 Apr 16;16:12589. doi: 10.1038/s41598-026-48476-5 (PMC13087045; doi:10.1038/s41598-026-48476-5)
Supplement: Supplementary file 1 — Supplementary Material 1 [file 41598_2026_48476_MOESM1_ESM.docx]

**Supplementary materials**

*Kozik et al., Longitudinal stability of cognitive impairments in post-COVID-19 syndrome assessed with the tablet-based Oxford Cognitive Screen-Plus*

Table of Contents

[Table S1: Oxford Cognitive Screen-Plus tasks descriptions 2](#_Toc220680807)

[Table S2: Sociodemographic and baseline clinical variables in Follow-Up vs. Non-Follow-Up patients 3](#_Toc220680808)

[Table S3: Performance on the Oxford Cognitive Screen-Plus composite scores between Non-Follow-Up (NFU) and Follow-Up (FU) patients at baseline visit 4](#_Toc220680809)

[Table S4: Results from multiple linear regressions investigating the associations between fatigue and depression and cognitive performance on the Oxford Cognitive Screen-Plus 5](#_Toc220680810)

# **Table S1: Oxford Cognitive Screen-Plus tasks descriptions**

| **Task** | **Description** |  | **Score range** |
| --- | --- | --- | --- |
| Picture Naming | Participants name four images of low-frequency objects. |  | 0 – 4 |
| Semantics | Out of an array of four images, participants identify the correct object based on semantic category. |  | 0 – 4 |
| Orientation | Orientation in time and space is assed using four questions pertaining to the current date, location, and the current German chancellor. |  | 0 – 4 |
| Word Memory Encoding | Participants are tasked with remembering a list of five words, which is presented twice. After each presentation, participants are asked to recall the words (Encoding 1 and Encoding 2). |  | 0 – 5, for Encoding 1 and Encoding 2, each |
| Trails | In two baseline conditions, participants connect circles and squares in increasing and decreasing order of size, respectively. From this, the Processing Speed score is calculated as baseline time divided by baseline accuracy. |  | ∞ |
|  | The switching condition entails connecting circles and squares in a complex, alternating rule, with circles going up and squares going down in size. The Trails Executive Score is calculated as accuracy in the switching condition divided by accuracy in the baseline condition. |  | 0 – 100 |
| Delayed Recall | After the Trails task, participants are asked to recall the encoded words (Delayed Recall). |  | 0 – 5 |
|  | Words, which were not correctly recalled, are presented as part of a multiple-choice array (Delayed Recall and Recognition). |  | 0 – 5 |
| Episodic Recognition | Participants select stimuli (objects or words), which were part of previous tasks out of a multiple-choice array. |  | 0 – 4 |
| Figure Copy | Participants copy a complex figure consisting of 20 geometric elements, each being scored for presence, position, and accuracy. |  | 0 – 60 |
|  | Participants are briefly presented with the same figure again, which then has to be drawn from memory. |  | 0 – 60 |
| Cancellation | Participants are presented with a search array of 60 pictograms of fruit (targets) and vegetables (distractors). Each of the 30 targets needs to be selected once and in the visible condition, selected elements are visibly marked. |  | 0 – 30 (accuracy) and 0 – ∞ (false positive selections) |
|  | In the invisible condition, the pictograms are re-arranged and presented again. Again, each target needs to be selected once, but in the invisible condition, the markings disappear immediately after selection. |  | 0 – 30 (accuracy) and 0 – ∞ (correct revisits) |

*Note.* this table has been previously published in Kozik et al. (2023)

**Table S2: Sociodemographic and baseline clinical variables in Follow-Up vs. Non-Follow-Up patients**

|  | **Distribution** | | | | **Estimate/ test statistic** | **p-value** | **Effect size** | **95% CI** |
| --- | --- | --- | --- | --- | --- | --- | --- | --- |
|  | **Follow-up** | | **Non-follow-up** | |  |  |  |  |
| Age  years, mean (SD, range) | 47.6 | (10.4, 22–64) | 46.5 | (11.7, 18 –65) | 7895^a^ | 0.692 | -0.024^c^ | [-0.132; 0.09] |
| Sex, female / male  No. (%) | 52 / 29 | (64 / 36) | 135 / 66 | (67 / 33) | 0.88^b^ | 0.677 | 0.877^d^ | [0.495; 1.571] |
| Hospitalisation  No., (%) | 24 | (30) | 43 | (21.4) | 1.73^b^ | 0.164 | 1.545^d^ | [0.82; 2.872]] |
| ICU ward  No. (%) | 8 | (33.3) | 14 | (7) | 0.309^b^ | 0.465 | 1.44^d^ | [0.503; 3.88] |
| Fatigue (FAS)  raw score, mean (SD, range) | 30.9 | (8.6, 13–50) | 31.4 | (9.2, 11 - 48) | 8331.5^a^ | 0.758 | 0.018^c^ | [-0.098; 0.132] |
| Depression (PHQ-9)  depression, raw score, mean (SD, range) | 10.7 | (4.5, 1–23) | 10.7 | (6, 0 - 29) | 7904.5^a^ | 0.704 | -0.023^c^ | [-0.135; 0.087] |

Note. SD = standard deviation; FAS = Fatigue Assessment Scale; PHQ-9 = Patient Health Questionnaire, depression module; ^a^ = Wilcoxon rank sum test (*W*); ^b^ = Fisher’s exact test (OR); ^c^ = rank biserial correlation; ^d^ = odd’s ratio; 95 % CI = 95% confidence interval.

# **Table S3: Performance on the Oxford Cognitive Screen-Plus composite scores between Non-Follow-Up (NFU) and Follow-Up (FU) patients at baseline visit**

|  | **Follow-up** | | | **Non-follow-up** | | | **Wilcoxon rank sum test** | | **Effect size^a^** | **95% CI** |
| --- | --- | --- | --- | --- | --- | --- | --- | --- | --- | --- |
| Composite score | n | M (SD) | Q2 (Q1, Q3) | n | M (SD) | Q2 (Q1, Q3) | W | *p* |  |  |
| Naming and Semantic Understanding | 79 | 7.81 (0.43) | 8 (8;8) | 201 | 7.84 (0.44) | 8 (8;8) | - | - |  |  |
| Memory Encoding | 79 | 9.52 (0.7) | 10 (9;10) | 192 | 9.43 (0.77) | 10 (9;10) | 7178.5 | .431 | -0.047 | [-0.155; 0.081] |
| Delayed Memory | 81 | 7.77 (1.71) | 8 (7;9) | 198 | 8.07 (1.79) | 8 (7;10) | 8931 | .129 | 0.091 | [-0.027; 0.198] |
| Attention | 79 | 57.99 (1.94) | 58 (57;59) | 196 | 58.32 (1.62) | 59 (57;60) | 8369 | .282 | 0.064 | [-0.044; 0.188] |
| Executive Functioning | 79 | 84.92 (23.83) | 100 (78.57;100) | 196 | 80.24 (25.1) | 91.67 (71.18;100) | 6660 | .058 | -0.113 | [-0.226; >0] |
| Praxis | 81 | 101.42 (11.54) | 104 (96;110) | 200 | 103.19 (9.31) | 105 (98;110) | 8584 | .432 | 0.047 | [-0.064; 0.177] |

*Note*. M = mean, SD = standard deviation, Q1, 2, 3 = quantiles 1, 2, 3; ^a^ = biserial rank correlation; 95 % CI = 95% confidence interval.

# **Table S4: Results from multiple linear regressions investigating the associations between fatigue and depression and cognitive performance on the Oxford Cognitive Screen-Plus**

| **Composite score** | **Predictor** | **Estimate (95 % CI)** | **Std. Error** | **t** | ***p*** | ***F*** | **df** | **R^2^** |
| --- | --- | --- | --- | --- | --- | --- | --- | --- |
| Delayed Memory | Intercept | -0.02 (-0.56, 0.39) | 0.23 | -0.07 | 0.94 |  |  |  |
|  | FAS delta | -0.04 (-0.13, 0.04) | 0.03 | -1.24 | 0.22 |  |  |  |
|  | PHQ-9 delta | -0.09 (-0.19, -0.01) | 0.06 | -1.56 | 0.12 |  |  |  |
|  |  |  |  |  |  | 2.74 | 2, 75 | 0.07 |
|  |  |  |  |  |  |  |  |  |
| Executive Functioning | Intercept | -1.25 (-10.16, 9.08) | 4.83 | -0.26 | 0.80 |  |  |  |
|  | FAS delta | -0.36 (-1.47, 1.11) | 0.55 | -0.66 | 0.51 |  |  |  |
|  | PHQ-9 delta | 0.7 (-2.28, 2.88) | 1.09 | 0.64 | 0.53 |  |  |  |
|  |  |  |  |  |  | 0.34 | 2, 50 | 0.01 |
|  |  |  |  |  |  |  |  |  |
| Attention | Intercept | -0.34 (-0.9, 0.15) | 0.30 | -1.15 | 0.25 |  |  |  |
|  | FAS delta | -0.03 (-0.09, 0.03) | 0.04 | -0.90 | 0.37 |  |  |  |
|  | PHQ-9 delta | 0.03 (-0.11, 0.14) | 0.07 | 0.35 | 0.73 |  |  |  |
|  |  |  |  |  |  | 0.41 | 2, 71 | 0.01 |

Note. CI = confidence intervals, Std. Error = standard error, delta = difference between visit 2 and baseline scores
